# Inter and intraspecies comparison of the level of selected bacterial phyla in in cattle and sheep based on feces

**DOI:** 10.1186/s12917-021-02922-w

**Published:** 2021-06-25

**Authors:** Natalia Szeligowska, Paulina Cholewińska, Katarzyna Czyż, Konrad Wojnarowski, Marzena Janczak

**Affiliations:** Institute of Animal Breeding, Wroclaw University of Environmental and Life Sciences, 51-630 Wroclaw, Poland

**Keywords:** Microbiome, Ruminants, Cattle, Sheep, Digestive tract, Genetic factors

## Abstract

**Background:**

The microbiome of the digestive tract of ruminants contains microbial ecosystem that is affected by both environmental and genetic factors. The subject of this study concerns the influence of selected genetic factors, such as species of animals and “host” individual differences on the digestive tract microbiome composition. The results show the core microbiological composition (Firmicutes and Bacteroidetes) of ruminants digestive tract (based on feces) depending on breed and “host”. The Bacteroidetes and Firmicutes phyla are the most abundant in ruminants digestive tract. The aim of the study was to determine the differences prevalence level of Bacteroidetes and Firmicutes phyla in feces of Charolaise cattle and Polish Olkuska Sheep with respect to intra- and inter-species variability.

**Results:**

The research group in the experiment consisted of animals at the age of 3 months kept in the same environmental conditions – rams of Polish Olkuska Sheep (*n* = 10) and Charolaise bulls (*n* = 10). Feces were collected individually from each animal (animals without disease symptoms were selected), living on the same environmental conditions. The analysis of the results in terms of species showed differences in the Firmicutes phylum level and *Lactobacillaceae* family between rams and bulls. Subsequently, the analysis performed for the “host effect” showed differentiation in the levels of the Bacteroidetes and Firmicutes phyla between individuals in a group and also between the groups.

**Conclusion:**

The obtained results suggest that, apart from the diet and the environment, the species and the individual host are equally important factors influencing the microbiological composition of the digestive system of ruminants.

## Introduction

Ruminants, including both sheep and cattle, are characterized by a high level of digestive system complexity, which is related to the way of digesting plants, as well as to the four-chamber structure of the stomach (rumen, omasum, abomasum, reticulum) [1–3]. The microbiome, a complex microbial community, is crucial for the health of ruminants, but also their productivity as well as improvement of animal based products. The microbiome inhabiting the digestive tract helps to maintain the body’s homeostasis and stimulate the immune system. Microorganisms, which include bacteria, viruses, fungi, archaea and protozoa, take part primarily in digestion processes. They ferment and decompose plant components (cellulose, hemicellulose, pectin) and volatile fatty acids (VFAs), which are further used by the host as a source of energy, involved in vitamin production, detoxification of toxic compounds etc. [3–6].

Both the quantity and the diversity of digestive tract of microflora can be influenced by environmental and genetic factors, such as age, breed, diet, heat stress, lactation period and sex hormones, animal maintenance conditions, and geographical location. It also varies in different parts of the digestive system. The most numerous bacteria inhabit the forestomachs and the large intestine, where the processes of fermentation or decomposition of nutrients take place. The dominant bacterial groups that colonize the digestive system of ruminants are Bacteroidetes and Firmicutes [6–10]. According to Wang et al. [7] and Mamun et al. [8] these bacteria the so-called core microbiome, which constitutes from 50 to 70% of the total number of microorganisms.

The influence of genetic factors on the microbiome of the digestive system of animals is related to the selection carried out in order to obtain products of interest to breeders (meat, milk, wool or leather), as well as to which the livestock system is adapted to the animals (pasture system, pasture and pasture system, etc.). In addition, due to the large number of ruminant species, ranging from cattle, to sheep and fallow deer, it is also important to understand the differences between them not only at the anatomical or physiological level, but also microbiological, in order to understand the operation of such a complex relationship that exists between ruminants. and their microbiome. In addition, recently the “host” factor, i.e., the direct influence of an individual on its microbiome composition, has been discussed more and more frequently as a genetic factor, however, these studies are most often performed in the case of humans rather than animals [9–12].

The aim of the study was to determine the level of selected bacterial phyla, i.e., Firmicutes and Bacteroidetes, and the level of the *Lactobacillaceae* family (Firmicutes phylum) depending on the species (Charolaise cattle and Okluska Sheep). In addition, the differences in the levels of the studied phyla in the studied individuals were analyzed.

## Materials and methods

### Animals

The animals used in the experiment included Charolaise cattle and Polish Olkuska sheep kept in the same environmental conditions, in the Research and Didactic Station in Swojec belonging to Wroclaw University of Environmental and Life Sciences (Poland), were reproduction of animals at the station was controlled in order to maintain the purity of the breed.

Charolaise is a beef cattle breed, characterized by maturation at the age of 18–24 months and fast growth rate (daily gains at the level of 1.5 kg / fattening period) [12].

Polish Olkuska sheep is a breed characterized by high prolificacy rate, high fertility (about 200%) and a good maternal instinct, and is also used for the crossbreeding with meat breed. In 2005, it was included in the Genetic Resources Protection Program due to the responsible gene for high prolificacy and good adaptation to the environmental conditions in Poland [13].

Ten individuals of cattle and sheep (male) at the age of 3 months were selected for the study. The animals selected for the experiment had to be in good health (no disease symptoms) for the period of stay at the Research and Didactic Station Swojec, as well as for a period of about a month after sampling, so that the samples came only from healthy animals with good condition and production indicators, so that the results are not disturbed. Therefore, such a restrictive approach resulted in the selection of 10 representative animals (10 cattle, 10 rams) from the flocks covered by the experiment. Additionally, the animals were kept in the same system and climatic conditions and there were kept under constant veterinary care. From birth to the experiment, the animals were not subjected to any zootechnical and veterinary treatments, i.e., vaccinations, deworming in order to eliminate additional factors that could affect the microbiome of the digestive system.

### Diet in the studied groups of animals

The sheep were allowed to graze from the 42nd day of life, they spent about 8 h a day on the pasture (with access to water). During this period, they received an addition of concentrated feed (oat grain) at the level of 70 g / head / day and had ad libitum access to hay. The feed dose was consistent with the INRA (Institut National de la Recherche Agronomique) standards [14].

In the same period, cattle received meadow hay ad libitum and alfalfa haylage (3 kg / head / day). The components and their quantity were determined in accordance with INRA standards [14].

All animals had ad libitum access to salt licks supplemented with Selenium.

All feeds provided to the animals during the experiment came from the same environment (same batch/farm, store).

### Samples collection

Fecal samples were collected individually from each examined animal up to 10 s after defecation into sterile containers (100 ml) (biological samples were collected twice – morning and evening, once), transported to the laboratory in a thermal container (at − 5 °C, 15 min), then frozen to − 26 °C until analysis (20 days) [15].

### Isolation of DNA

The Genomic Mini AX Stool kit (A&A Biotechnology, Gdańsk, Poland) was used for DNA isolation, and it was modified by the addition of mutanolysin and lysozyme.

After isolation, the quality of the DNA obtained was verified using a NanoDrop 2000 spectrophotometer from Thermo Scientific (Wilmington, NC, USA). The average DNA content was 80–100 μg/μL. The level of impurities in the samples was 2.0–2.2 for parameter 260/230: and 1.8–2.0 for parameter 260/280. In the case of high levels of impurities or possibly low-quality DNA, the samples were re-isolated or cleaned with the Clean-up Concentrator (A&A Biotechnology, Gdańsk, Poland) [15]. The main problem in DNA isolation from fecal samples was the occurrence of various types of inhibitors that interfered with Taq polymerase and / or primer, which include causes a decrease in primer efficiency etc., more information Taylor et al. [16].

The isolation was carried out at approximate temperatures and humidity.

### Real-time PCR analysis

Real-time PCR analysis was performed with the use of a Bio-Rad CFX Connect 96 Touch apparatus with the SsoAdvanced™ Universal SYBR® Green Supermix kit (Bio-Rad Laboratories, Inc., CA, USA) at a volume of 10 μL in 3 technical repetitions (Table 1.). A no template control (NTC – without DNA sample, only primers and water with PCR mix) test was additionally performed for each gene. The real-time PCR analysis strategy was based on the amplification of genes specific for the tested phyla against the reference primer for all bacteria (16S). The reference primers were 16S universal eubacterial genes (Table 2) [15, 17–19].

**Table 1:** Mix Ratio to real – time PCR [15]

Component	Volume in a 10 μl reaction
SsoAdvanced™ Universal SYBR® Green Supermix	5 μl
Primer (F + R)	1 μl (0.8 μM)
DNA matrix	2 μl (0.04–0.015 × 10^−4^)
Sterile water	2 μl

**Table 2:** RT – PCR Primers [15, 17–19]

NAME	FORWARD (5′-3′)	REVERSE (5′-3′)
UNIVERSAL EUBACTERIAL GENES [17]	530F (5′-GTC CCA GCM GCN GCG G)	1100R (5′-GGG TTN CGN TCG TTG)
FIRMICUTES [18]	928F-Firm (5′-TGA AAC TYA AAG GAA TTG ACG)	1040FirmR (5′-ACC ATG CAC CAC CTG TC)
BACTEROIDETES [18]	798cfbF (5′-CRA ACA GGA TTA GAT ACC CT)	cfb967R (5′-GGT AAG GGT TCC TCG CGT AT)
LACTOBACILLACEAE [19]	lac1 forward (5′-AGC AGT AGG GAA TCT TCC A)	Lac2Seq (5′-ATTTCACCGCTACACATG)

To the performance of individual gene a standard curve was prepared for the primers. A sample dilution of 10^− 6^ from the 10^− 2^ to 10^− 7^ series of dilutions was selected for analysis. The analysis was performed according to a protocol of 40 cycles: polymerase activation and DNA denaturation 95 °C (3 min), denaturation 95 °C (15 s), annealing 60.5 °C (15 s), extension and plate reading at 72 °C (40 s). The analysis of the melting curves for the samples was performed at temperatures ranging from 65 °C (5 s) to 95 °C (0.5 °C increments in 2 s). The sample with a DNA level of 100 μg/μL and impurities at a level in line with the above mentioned standards was an arbitrary calibrator [15].

The efficiency of individual primers was normal (according to the standards established by BIO - RAD) and amounted to 87.4% for Firmicutes, 103% for Bacteroidetes, 97.7% for *Lactobacillaceae*. Universal primer efficiency was 94.4% for both cattle and sheep fecal samples.

The RT PCR results data was processed using the CFX Maestro software (Bio-Rad Laboratories, Inc., California), where the sample with a DNA quantity of 40 μg / μl and impurity levels compliant with the above standards was an arbitrary calibrator. The obtained results were calculated by the CFX Maestro program in relation to the amount of reference primer template and differences at the level of the studied phyla and families genes. Relative Normalized Expression (ΔΔCq - relative normalized expression calculated using control samples and reference targets), taking into account the amplification efficiency of individual primers, indicated the level of the studied phyla in the collected samples [20, 21].

### Statistical analysis

The obtained results were analyzed using the Statistica ver. 13.1 (Statsoft, Poland). The data distribution was checked with the Shaphiro-Wilk test. Due to the lack of a normal distribution (RNE results for cattle and sheep), the Mann-Whitney U test (*P* > 0.05) was used.

## Results

**Fig. 1:**
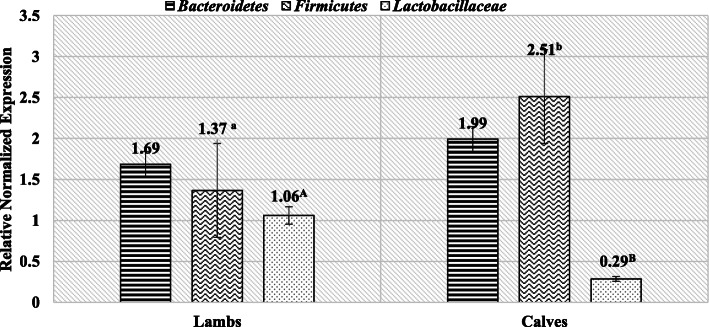
Level of RNE of selected phyla and family in rams and bulls feces (*p* > 0.05 – **a**, **b**; *p* > 0.01 – **A**, **B**)

The results of the rt-PCR analysis showed significant differences in the RNE (Relative Normalized Expression) level of the Firmicutes phylum (*p* = 0.048) and highly significant in the level of the *Lactobacillaceae* family (*p* = 0.00017) between the lambs and bulls. No differences in the level of the Bacteroidetes phylum were found between the studied ruminant species, however, the level of this phylum tended to increase in the examined bulls compared to the lambs (Fig. 1). Much higher, about 2-fold, levels of the Firmicutes phylum were also found in male bulls compared to lambs (RNE 2.51 and 1.37, respectively). Additionally, the ratio of Firmicutes to Bacteroidetes was opposite in lambs compared to cattle, in lambs the majority was represented by Bacteroidetes, while in bulls by the Firmicutes phylum (Table 3).

**Table 3:** Individuals level of selected bacteria phyla in calves and lambs feces (*p* > 0.05 – a, b)

PHYLA	BACTEROIDETES				FIRMICUTES			
Number	LAMBS		CALVES		LAMBS		CALVES	
	Average (RNE)	*SD*	Average (RNE)	*SD*	Average (RNE)	*SD*	Average (RNE)	*SD*
1	0.88	*0.05*	1.22	*0.21*	0.34	*0.01*	2.36	*0.36*
2	3.72	*0.12*	1.62	*0.24*	1.23	*0.09*	2.64	*0.91*
3	1.31	*0.32*	2.11	*0.99*	1.23	*0.22*	0.8	*0.11*
4	3.47	*0.52*	1.16	*0.23*	1.21	*0.56*	0.15	*0.01*
5	4.58	*0.58*	2.62	*0.56*	3.32	*0.96*	3.71	*1.11*
6	0.51	*0.01*	2.29	*0.85*	0.55	*0.026*	1.08	*0.85*
7	1.25	*0.60*	0.70	*0.06*	2.78	*0.25*	1.59	*0.47*
8	0.77	*0.01*	5.81	*0.98*	2.07	*0.75*	8.76	*1.20*
9	0.12	*0.01*	0.79	*0.03*	0.21	*0.01*	1.71	*0.69*
10	0.24	*0.08*	1.57	*0.32*	0.71	*0.06*	2.26	*0.58*
Average (RNE)	1.69		1.99		1.37^a^		2.51^b^	
*SD*	*1.53*		*1.41*		*0.99*		*2.29*	

**Fig. 2:**
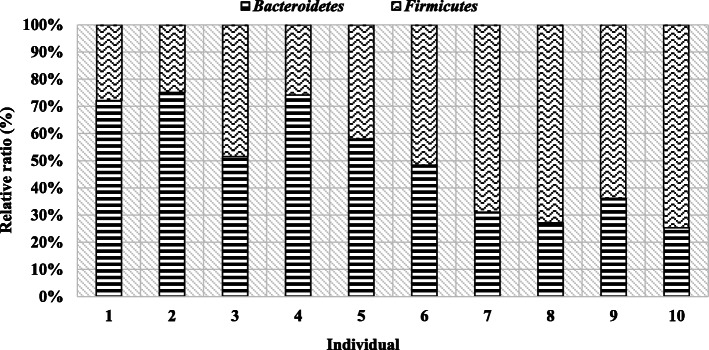
Individual bacteria differences in rams feces

**Fig. 3:**
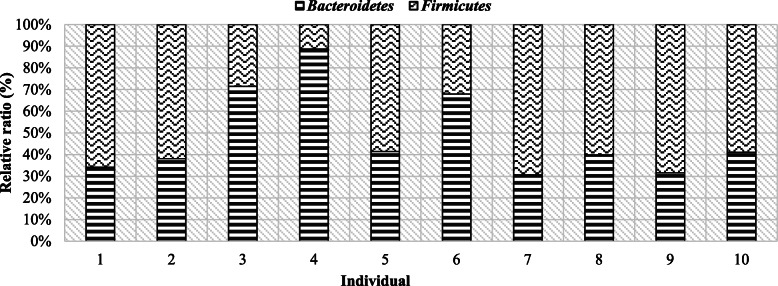
Individual bacteria differences in bulls feces

In order to present intra-species differences in terms of microbiome composition, comparisons of the occurrence of individual bacterial phyla in the studied individuals were made (Table 3, Figs. 2 and 3).

The results show the difference between individuals, both bulls and rams, in terms of relative averages for *Bacteroidetes* and *Firmicutes* phyla. In the case of lambs, Bacteroidetes phylum had a greater share in five individuals, n. 1–5 (Table 3, Fig. 2). The opposite situation was noted in lambs no. from 6 to 10, in their case, the larger group was represented by Firmicutes (more than 50%). However, despite individual differences, some of the lambs showed a similar level of the studied groups, e.g. individuals 1, 2 and 4, or 7, 8 and 10. Individuals 1, 2 and 4 had a higher proportion of the Bacteroidetes phylum (more than 65%) compared to Firmicutes. Then, in the case of individuals from 7 to 10, the Firmicutes phylum was predominant (more than 60%).

**Fig. 4:**
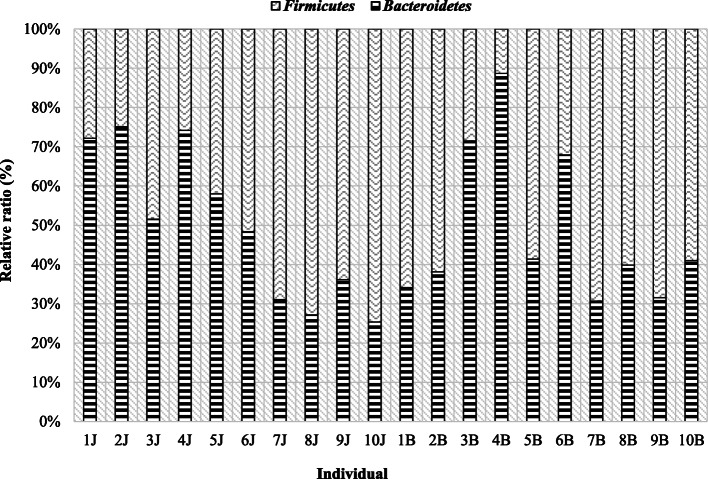
Comparison of the level of studied phyla in rams and bulls feces

The occurrence of intra-species differentiation can also be noted on the example of bulls, although there were also similarities, e.g. between individuals 2, 8, 10, which were characterized by a higher share of the Firmicutes phylum (Table 3, Fig. 3). In seven individuals, no. 1, 2, 5, 7–10, Firmicutes phylum had a greater share compared to Bacteroidetes, while in lambs the same situation was found in half of the individuals. However, both in male bulls and lambs, individual differences are visible not only within the species but also between the species, which is also shown in Fig. 4.

The level of *Lactobacillaceae* family in the individual animals of both species is presented in Table 4. It was more that 3-fold higher in lambs compared to bulls, and some intra-species differentiation can be found.

**Table 4:** Individual level of *Lactobacillaceae* in lambs and calves feces (*p* > 0.01 – A, B)

Number	*Lactobacillaceae*			
	LAMBS		CALVES	
	Average (RNE)	*SD*	Average (RNE)	*SD*
1	1.01	*0.17*	0.07	*0.00*
2	1.75	*0.65*	0.01	*0.00*
3	1.15	*0.17*	1.75	*0.21*
4	0.93	*0.05*	0.05	*0.03*
5	1.53	*0.25*	0.03	*0.00*
6	0.64	*0.04*	0.08	*0.00*
7	1.23	*0.59*	0.57	*0.12*
8	0.96	*0.15*	0.03	*0.01*
9	0.51	*0.10*	0.05	*0.01*
10	0.91	*0.25*	0.23	*0.10*
Average (RNE)	1.06^A^		0.29^B^	
*SD*	*0.36*		*0.51*	

## Discussion

The microbiome influences many physiological aspects of the host, in both humans and animals, conferring traits that would not have developed without their participation, such as digestion of plant-based fodder in the case of ruminants. Ruminants differ, as do humans, biologically or genetically, which in turn can be considered further factors that differentiate microbial composition in the gastrointestinal tract [6, 22]. Each individual also has its own individual microbiome, which is increasingly considered a phenotypic characteristic of the animal [1, 23].

The microbiome - digestive system - host relationship affects the development and proper health status of the animal (without any symptoms of sickness and maintained in proper welfare). In the case of ruminants, the rumen is the most extensive part of the digestive system in terms of microbiology, and the large intestine is another site highly colonized by microorganisms. In the final part of the digestive system, starting from the large intestine, the count of bacteria increases in the caudal direction [24, 25]. Additionally, changes in the composition of the microbiome can be observed both in the feces and in the rumen [26].

The study by Xin et al. [27] showed that despite a similar diet or the same environment, species plays an important role in influencing the composition of the gastrointestinal tract of ruminants, similar results were also presented by Henderson et al. [1]. The authors of the above studies [1, 27] suggest that differences in the microbial composition of the gastrointestinal tract may be due to the process of animals adaptation to the environment. On the other hand, recent studies on microbial differences in the gastrointestinal tract between sheep breeds suggest that factors such as geographic origin and the nature of animal breed utilization may influence the microbiome composition within the breed [28–30]. The obtained results of real time PCR analysis indicate that despite being in the same area (including fed similarly), bulls and rams differed in the level of Firmicutes phylum, including the *Lactobacillaceae* family. Diet may have been a factor further influencing the ruminant microbiome, in addition to genetics. During this period, lambs were fed mainly pasture greens with the addition of concentrate feed (oats) and hay ad libitum, while the bulls had no access to fresh greens. However, the amount of concentrate feed did not exceed 30% of the daily ration in both rams and bulls. For ruminants, the amount of concentrate feed is an important factor affecting microbiome composition, as its increased proportion can significantly affect the levels of microbial groups studied. Moreover, it was demonstrated that Firmicutes and Bacteroidetes phyla are correlated, i.e., an increase in one phylum level is accompanied by a decrease in the second one [31]. Increased amount of concentrate feed in ruminants could have contributed to higher levels of Firmicutes compared to Bacteroidetes, however, in this study, the animals received a low concentrated feed with a small addition of concentrate feed, which probably did not have a significant effect on the levels of the studied phyla [2, 8, 27]. Therefore, in this study, it can be suggested that mainly the genetic factor, i.e., species, had a significant effect on the gastrointestinal microbiome.

The differences in the level of the *Lactobacillaceae* family in male bulls compared to rams, may indicate that bull’s rumen was probably more developed at 3 months of age compared to rams. Intensive fermentation and a significant development of the microbiome in the rumen begins with the beginning of solid feed consumption. During this time, microorganisms from the feed enter the rumen. Nevertheless, some of the microorganisms that inhabit the digestive system begin to function and proliferate after parturition, which was presented in the studies by Jami et al. [28] and Li et al. [29]. The amount and activity of microorganisms in the digestive system changes with the age of the animals. The analysis of the digestive system of cattle showed that in the early stages of life, aerobic and facultatively anaerobic bacteria, e.g. from the *Lactobacillaceae* family, are more abundant and more active. In digestive tract of 6–8 weeks old calves, the number of relatively anaerobic bacteria decreases in favor of anaerobic ones. The microbiological composition of the digestive system in young animals changes and stabilizes until the 83rd day of life [24, 30]. Therefore, the lambs which had unlimited access to mother’s milk until day 40 postpartum, and during this period it constituted the majority of their diet could have been characterized by a higher level of the studied *Lactobacillaceae* family, which may indicate further development of their rumen. On the other hand, the bulls were mainly on solid forage from 2 weeks of age, which could have contributed to faster rumen development compared to rams [6, 24, 32]. It was also demonstrated that the composition of rumen, and thus feces, microbiome is determined by the feed consumed, i.e., gram-negative bacteria dominate when animals are fed high forage diet, while gram-positive ones, like *Lactobacillaceae,* in the case of diet rich in grains [33]. Moreover, according to Chen et al. [33], the changes in the diet may cause a decrease in rumen pH and consequently an increase in *Lactobacillaceae* count, which can in part explain higher level of this family in sheep, who changed their diet from mother milk to pasture green.

Many microbiome studies also suggest that individual or intra-species influences, the so-called “host influence”, can have a large impact on the levels of bacteria in the gastrointestinal tract. In human studies conducted by the Human Microbiome Project [24], researchers suggest that each individual has its own gastrointestinal microbial composition; similar findings are often described even in ruminants, such as in the study by Lopes et al. [26]. According to Furman et al. [34], the composition of microbiome in younger individuals is more differentiated within the species than in older ones, which may suggest partly random invasion of microbial species at early stages on animal development.

The study conducted by Turnbaugh et al. [24] on the human microbiome showed that it is shared partially by family members, however, there are individual differences. The results obtained suggest that a common microbiota is only present when the population is small. However, the study by Qin et al. [35], made it possible to delineate common microorganisms at the species level in a continental (European) population. This study on 124 Europeans showed that 18 bacterial species were present in all samples and 57 in 90% of individuals from the same population, covering the European region. Similar relationships have also been shown for ruminants. The occurrence of core microbial composition was confirmed in the study by Lopes et al. [26] or Wang et al. [7]. In the studies conducted by the aforementioned authors, it was shown that the main phyla inhabiting the gastrointestinal tract are Firmicutes and Bacteroidetes. Their population represents on average 50% of the total number of bacteria. However, the study conducted by Lopes et al. [26] also showed the presence of individual differences. The sheep studied differed mainly in the ratio of Firmicutes to Bacteroidetes. Conclusions similar to the work of Lopes et al. [26] are also suggested by Mamun et al. [8], who documented the presence of a core microbiome, i.e., Firmicutes and Bacteroidetes phyla as well as the presence of individual differences between the sheep studied. In this experiment, the core microbial population was in turn demonstrated at a higher level of over 70%. The results obtained in our study indicate that the individual microbiome varies in both rams and bulls. It can be observed that the studied animals, despite similar nutrition, environment or even genetics, differed within the group, which may indicate the influence of the individual on the microbial composition. Similar relationships were also described in studies by Mamun et al. [8] or Zhang et al. [36] who showed individual differences between the sheep studied. However, this issue needs further analysis in ruminants [37, 38].

## Conclusions

The study showed inter-species differences in the certain species of bacteria of the digestive system. Additionally, individual, i.e., intra-species differences were found in the studied groups of animals. The obtained results suggest that there are differences in the microbiological composition of the digestive system of ruminants not only in terms of diet or environment, but also genetics. Additionally, the study also focused on the “host influence”, showing that despite the same environmental conditions, similar diet, origin and breed, significant differences between the individuals, both in the group of rams and bulls may be observed. However, further research on changes in the microbiome depending on species and individual host is recommended.

## Data Availability

The datasets generated and/or analyzed during the current study are not publicly available because a part of them belong to a different set of studies (for personal use), but are available from the corresponding author on reasonable request.

## Abbreviations

VFA: Volatile Fatty Acids
DNA: Deoxyribonucleic acid
PCR: Polymerase Chain Reaction
NTC: No Template Control
INRA: Institut National de la Recherche Agronomique
RNE: Relative Normalized Expression

## References

[1] Henderson G, Cox F, Ganesh S, Jonker A, Young W, Collaborators GRC, Janssen PH (2015). Rumen microbial community composition varies with diet and host, but a core microbiome is found across a wide geographical range. Sci Rep.

[2] Khafipour E, Li S, Tun H, Derakhshani H, Moossavi S, Plaizier J (2016). Effects of grain feeding on microbiota in the digestive tract of cattle. Anim Front.

[3] O'Hara E, Neves AL, Song Y, Guan LL (2020). The role of the gut microbiome in cattle production and health: driver or passenger?. Annual Rev Anim Biosci.

[4] Opdahl LJ, Gonda MG, St-Pierre B (2018). Identification of uncultured bacterial species from *Firmicutes*, *Bacteroidetes* and *Candidatus saccharibacteria* as Candidate cellulose utilizers from the rumen of beef cows. Microorganisms.

[5] Tanca A, Fraumene C, Manghina V, Palomba A, Abbondio M, Deligios M, Uzzau S (2017). Diversity and functions of the sheep faecal microbiota: a multi-omic characterization. Microb Biotechnol.

[6] Cholewińska P, Czyż K, Nowakowski P, Wyrostek A (2020). The microbiome of the digestive system of ruminants – a review. Anim Health Res Rev.

[7] Wang L, Zhang K, Zhang C, Feng Y, Zhang X, Wang X, Wu G (2019). Dynamics and stabilization of the rumen microbiome in yearling Tibetan sheep. Sci Rep.

[8] Mamun MAA, Sandeman M, Rayment P, Brook-Carter P, Scholes E, Kasinadhuni N, Greenhill AR (2020). The composition and stability of the faecal microbiota of merino sheep. J Appl Microbiol.

[9] Lima J, Auffret MD, Stewart RD, Dewhurst RJ, Duthie C-A, Snelling TJ, Walker AW, Freeman TC, Watson M, Roehe R (2019). Indetification od rumen microbial genes involved in pathways linked to appetite, growth and feed conversion efficiency in cattle. Front Genet.

[10] Myer PR, Smith TPL, Wells JE, Kuehn LA, Freetly HC (2015). Rumen microbiome from steers differing in feed efficiency. PLoS One.

[11] Li F, Guan LL (2017). Metatranscriptomic profiling reveals linkages between the active rumen microbiome and feed efficiency in beef cattle. Appl Environ Microbiol.

[12] Cholewińska P, Iwaszkiewicz M, Łuczycka D, Wysoczański T, Nowakowski P, Czyż K, Wyrostek A, Bodkowski R (2019). Electrical characteristics based on resistance and impedance of polish Olkuska sheep lambs wool. J Nat Fibers.

[13] Polski Związek Owczarski. http://pzow.pl/. Accessed 15 Jan 2021.

[14] Strzetelcki JA, Brzóska F, Kowalski ZM, Osięgłowski S (2014). Zalecenia żywieniowe dla przeżuwaczy i tabele wartości pokarmowej pasz.

[15] Cholewińska P, Wołoszyńska M, Michalak M, Czyż K, Rant W, Janczak M (2020). Evaluation of Changes in the Levels of Firmicutes and Bacteroidetes Phyla of Sheep Feces Depending on the Breed. Animals.

[16] Taylor SC, Laperriere G, Germain H (2017). Droplet Digital PCR versus qPCR for gene expression analysis with low abundant targets: from variable nonsense to publication quality data. Sci Rep.

[17] Dowd SE, Callaway TR, Wolcott RD, Sun Y, McKeehan T, Hagevoort RG, Edrington TS (2008). Evaluation of the bacterial diversity in the feces of cattle using 16S rDNA bacterial tag-encoded FLX amplicon pyrosequencing (bTEFAP). BMC Microbiol.

[18] De Gregoris TB, Aldred N, Clare AS, Burgess JG (2011). Improvement of phylum-and class-specific primers for real-time PCR quantification of bacterial taxa. J Microbiol Methods.

[19] Walter J, Hertel C, Tannock GW, Lis CM, Munro K, Hammes WP (2001). Detection of *Lactobacillus, Pediococcus, Leuconostoc*, and *Weissella* species in human feces by using group-specific PCR primers and denaturing gradient gel electrophoresis. Appl Environ Microbiol.

[20] Rocha DJ, Castro TL, Aguiar ER, Pacheco LG (2020). Gene expression analysis in Bacteria by RT-qPCR. Methods Mol Biol.

[21] CFX Maestro Software. User Guide Version 1.1. Bio-Rad. https://www.bio-rad.com/webroot/web/pdf/lsr/literature/10000068703.pdf . Accessed 28 Nov 2020.

[22] Meale SJ, Li S, Azevedo P, Derakhshani H, Plaizier JC, Khafipour E, Steele MA (2016). Development of ruminal and fecal microbiomes are affected by weaning but not weaning strategy in dairy calves. Front Microbiol.

[23] Malmuthuge N, Guan LL (2016). Gut microbiome and omics: a new definition to ruminant production and health. Anim Front.

[24] Turnbaugh PJ, Ley RE, Hamady M, Fraser-Liggett CM, Knight R, Gordon JI (2007). The human microbiome project. Nature..

[25] Plaizier JC, Li S, Danscher AM, Derakshani H, Andersen PH, Khafipour E (2017). Changes in microbiota in rumen digesta and feces due to a grain-based subacute ruminal acidosis (SARA) challenge. Microb Ecol.

[26] Lopes LD, De Souza Lima AO, Taketani RG, Darias P, Da Silva LRF, Romagnoli EM, Mendes R (2015). Exploring the sheep rumen microbiome for carbohydrate-active enzymes. Antonie Van Leeuwenhoek.

[27] Xin J, Chai Z, Zhang C, Zhang Q, Zhu Y, Cao H, Ji Q (2019). Comparing the microbial community in four stomach of dairy cattle, yellow cattle and three yak herds in Qinghai-Tibetan plateau. Front Microbiol.

[28] Jami E, Israel A, Kotser A, Mizrahi I (2013). Exploring the bovine rumen bacterial community from birth to adulthood. The ISME Journal.

[29] Li M, Zhou M, Adamowicz E, Basarab JA (2012). Characterization of bovine ruminal epithelial bacterial communities using 16S rRNA sequencing, PCR-DGGE, and qRT-PCR analysis. Vet Microbiol.

[30] Malmuthuge N (2017). Understanding host-microbial interactions in rumen: searching the best opportunity for microbiota manipulation. J Anim Sci Biotechnol.

[31] Jami E, White BA, Mizrahi I (2014). Potential role of the bovine rumen microbiome in modulating milk composition and feed efficiency. PLoS One.

[32] Li Z, Wright AD, Liu H, Bao K, Zhang T, Wang K, Cui X, Yang F, Zhang Z, Li G (2015). Bacterial community composition and fermentation patterns in the rumen of sika deer (Cervus nippon) fed three different diets. Microb Ecol.

[33] Matthews C, Crispie F, Lewis E, Reid M, O’Toole PW, Cotter PD (2019). The rumen microbiome: a crucial consideration when optimising milk and meat production and nitrogen utilisation efficiency. Gut Microbes.

[34] Furman O, Shenhav L, Sasson G, Kokou F, Honig H, Jacoby S, Hertz T, Cordero OX, Halperin E, Mizrahi I (2020). Stochasticity constrained by deterministic effects of diet and age drive rumen microbiome assembly dynamics. Nat Commun.

[35] Qin J, Li R, Raes J, Arumugam M, Burgdorf KS, Manichanh C, Mende DR (2010). A human gut microbial gene catalogue established by metagenomic sequencing. Nature..

[36] Zhang H, Shao M, Huang H, Wang S, Ma L, Wang H, Zhu R (2018). The dynamic distribution of small-tail han sheep microbiota across different intestinal segments. Front Microbiol.

[37] Paster BJ, Russell JB, Yang CMJ, Chow JM, Woese CR, Tanner R (1993). Phylogeny of the ammonia-producing ruminal bacteria *Peptostreptococcus anaerobius*, *Clostridium sticklandii*, and *Clostridium aminophilum sp. nov*. Int J Syst Evol Microbiol.

[38] Chen Y, Oba M, Guan LL (2012). Variation of bacterial com-munities and expression of toll-like receptor genes in the rumen of steers differing in susceptibility to sub-acute ruminal acidosis. Vet Microbiol.

